# Associations of Organic Produce Consumption with Socioeconomic Status and the Local Food Environment: Multi-Ethnic Study of Atherosclerosis (MESA)

**DOI:** 10.1371/journal.pone.0069778

**Published:** 2013-07-31

**Authors:** Cynthia L. Curl, Shirley A. A. Beresford, Anjum Hajat, Joel D. Kaufman, Kari Moore, Jennifer A. Nettleton, Ana V. Diez-Roux

**Affiliations:** 1 Department of Environmental and Occupational Health Sciences, University of Washington, Seattle, Washington, United States of America; 2 Department of Epidemiology, University of Washington, Seattle, Washington, United States of America; 3 Departments of Environmental and Occupational Health Sciences, Epidemiology and Medicine, University of Washington, Seattle, Washington, United States of America; 4 Department of Epidemiology, University of Michigan, Ann Arbor, Michigan, United States of America; 5 Department of Epidemiology, University of Texas Health Science Center, Houston, Texas, United States of America; CUNY, United States of America

## Abstract

Neighborhood characteristics, such as healthy food availability, have been associated with consumption of healthy food. Little is known about the influence of the local food environment on other dietary choices, such as the decision to consume organic food. We analyzed the associations between organic produce consumption and demographic, socioeconomic and neighborhood characteristics in 4,064 participants aged 53–94 in the Multi-Ethnic Study of Atherosclerosis using log-binomial regression models. Participants were classified as consuming organic produce if they reported eating organic fruits and vegetables either “sometimes” or “often or always”. Women were 21% more likely to consume organic produce than men (confidence interval [CI]: 1.12–1.30), and the likelihood of organic produce consumption was 13% less with each additional 10 years of age (CI: 0.84–0.91). Participants with higher education were significantly more likely to consume organic produce (prevalence ratios [PR] were 1.05 with a high school education, 1.39 with a bachelor's degree and 1.68 with a graduate degree, with less than high school as the reference group [1.00]). Per capita household income was marginally associated with produce consumption (p = 0.06), with the highest income category more likely to consume organic produce. After adjustment for these individual factors, organic produce consumption was significantly associated with self-reported assessment of neighborhood produce availability (PR: 1.07, CI: 1.02–1.11), with an aggregated measure of community perception of the local food environment (PR: 1.08, CI: 1.00–1.17), and, to a lesser degree, with supermarket density (PR: 1.02: CI: 0.99–1.05). This research suggests that both individual-level characteristics and qualities of the local food environment are associated with having a diet that includes organic food.

## Introduction

The National Organic Program (NOP) of the United States Department of Agriculture permits food to be certified “organic” when grown without use of specified pesticides and synthetic fertilizers [1]. In the US, sales of organic food have grown steadily in the past two decades, from $1 billion in 1990 to $26.7 billion in 2010 [2].

Little research to date has examined the direct effect of organic food consumption on health [3], but several studies have shown that consumption of organic food, and particularly organic produce, can significantly reduce pesticide exposure [4]–[6]. The American Academy of Pediatrics recently released a report concluding that organic diets expose consumers to fewer pesticides associated with human disease [7]. This conclusion was based, in part, on several studies of pesticide exposure in children and pregnant women that suggest even relatively low exposures to certain agricultural pesticides may be associated with developmental and neurocognitive effects, such as decreased gestational age at birth and birth weight, and increased attention deficit-hyperactivity disorder and decrements in memory and IQ [8]–[13]. Choice of organic food is also an opportunity to support farming practices that can reduce risks to farmworkers and promote ecological health [7], [14].

Everyone may not have equal access to organic food, and thus may not have equal ability to make these choices. Organic food is more expensive that conventionally grown food, and it also may not be equally available in all communities. Research suggests that residents of neighborhoods with better access to healthy foods tend to have healthier diets [15]. We hypothesize a parallel in respect to organic food consumption, specifically that residents of neighborhoods with better access to organic food may be more likely to eat organic food.

The purpose of this study is to examine the relationship between organic produce consumption and individual demographic and socioeconomic factors including sex, race/ethnicity, age, income, education, metropolitan area and employment status in a multi-city, multi-ethnic cohort. We further explore the relationship between organic produce consumption and three complementary measures of the local food environment intended to represent food accessibility: 1) Geographic information system (GIS) based supermarket density, 2) self-reported assessments, and 3) aggregated survey responses of independent informants.

## Materials and Methods

This cross-sectional study investigates the organic produce consumption habits of participants in the Multi-Ethnic Study of Atherosclerosis (MESA). MESA was initiated in 1999 by the National Heart, Lung, and Blood Institute to investigate subclinical cardiovascular disease among 6,814 participants from six US areas: Baltimore City and Baltimore County, Maryland; Chicago, Illinois; Forsyth County, North Carolina; Los Angeles County, California; New York, New York; and St. Paul, Minnesota [16]. Participants were recruited using both random-digit dialing and brochures mailed to households in targeted areas, and were aged 45 to 84 years at enrollment with an approximately equal gender ratio. The MESA cohort is 39% Caucasian, 28% African American, 22% Hispanic, and 12% Chinese-American. The study was approved by the institutional review board at each site, and all subjects gave written informed consent. This includes the IRBs at UCLA, Columbia University, Johns Hopkins University, the University of Minnesota, Wake Forest University, and Northwestern University.

### Outcome Measures

Most data collection in MESA is structured around a series of clinical exams, scheduled at approximately two year intervals. The baseline exam occurred between July 2000 and July 2002, and the most recent exam, “Exam 5”, spanned April 2010 through February 2012. The analysis presented here primarily employs data collected at Exam 5. All participants attending this exam were asked to complete a food frequency questionnaire that inquired about eating habits over the previous year and included items about organic produce consumption. Specifically, participants were asked how often the fruit and vegetables they ate were “organically grown”, defined as “[having] a ‘USDA Organic’ label, purchased locally from an ‘organic farm’, or grown without pesticides in a home garden”. Options were “Seldom or Never”, “Sometimes”, and “Often or Always”. For the primary analysis, participants who reported that they sometimes, often or always ate either organic fruit or organic vegetables were categorized as “organic consumers”, and those who reported that they seldom or never ate organic fruit and organic vegetables were categorized as non-consumers. A separate, secondary analysis restricted the definition of organic consumers to just those who reported they “often or always” consumed organic fruit and vegetables.

### Individual-Level Variables

We hypothesized organic consumption to be associated with individual-level factors, including sex, age, race/ethnicity, metropolitan area, marital status, per capita income [total household income divided by number of persons living in the household], education, and employment status. With the exception of age, all variables were evaluated categorically.

### Neighborhood-Level Variables

We also hypothesized a relationship between organic consumption and a set of complementary measures of the local food environment, after control for individual-level variables. Specifically, we hypothesized organic produce to more likely be consumed by individuals living in areas with more supermarkets and where there is a perception of a larger selection of produce in general. These measures were developed by the MESA Neighborhood Study, an ancillary study to MESA that characterized the local food environments of MESA participants [17]–[18]. Each measure is briefly described here.

The first was a GIS-based measure representing the density of supermarkets within 1 mile of participants' homes. The density of supermarkets was determined using data obtained from the National Establishment Time Series (NETS) database from Walls and Associates [19]. Additional supermarket data was obtained from Nielsen/TDLinx to enhance the identification of supermarkets [20]. Supermarkets were defined as grocery stores (SIC code #5411) with at least $2 million in annual sales or at least 25 employees. Participant addresses were geocoded using TeleAtlas EZ-Locate web-based geocoding software [21], and simple densities per square mile were created for 1-mile buffers around each address using the point density command in ArcGIS 9.3.

The second measure was the participants' self-report of the selection of fruits and vegetables available in their neighborhoods, defined as the area within approximately 1 mile of their home (“MESA self-reports”). At Exam 5, participants were asked the extent to which they agreed with the statement “A large selection of fresh fruits and vegetables is available in my neighborhood”, and responses were coded on a five-point Likert scale (strongly agree; agree; neutral; disagree; and strongly disagree).

The third measure, the Aggregated Neighborhood Survey (ANS), was constructed by aggregating responses of multiple respondents residing in each participant's census tract (as a proxy for neighborhood). Survey respondents used in the calculation of the ANS included other MESA participants living within a given census tract as well as other residents in those census tracts who were included to increase the sample size in areas with few MESA respondents [22]. This supplementary survey was conducted on a random sample of residents in selected tracts, identified through address-based sampling methods. Availability of healthy food was ascertained based on responses to three survey items: “A large selection of fresh fruit and vegetables is available in my neighborhood”, “A large selection of low-fat food is available in my neighborhood”, and “The fresh fruits and vegetables in my neighborhood are of high quality”, with responses coded on five-point Likert scales. Conditional empirical Bayes estimates, which borrow information across all tracts in order to increase reliability, were derived from three level hierarchical linear models to account for the nested structure of the data [22].

### Statistical Analyses

For both individual and neighborhood analyses, we first conducted bivariate comparisons exploring the relationship between each variable and organic consumption, using either chi-squared tests or log-binomial regression, as appropriate. We then included the full set of individual-level variables in a log-binomial regression to model the association with organic consumption. Individual-level variables found to be statistically significant in the full model were included in the analyses of organic consumption and the local food environment. Log-binomial models were used in the primary analysis due to the relatively high prevalence of sometimes, often or always consuming organic produce (40%, n = 1,644). In the secondary analysis, where the outcome was the smaller set of individuals who reported they “often or always” consumed organic food (5%, n = 204), we employed logistic regression models.

The relationship between each measure of the local food environment and organic consumption was evaluated separately with and without control for individual-level variables. For each of these measures, we also examined the impact of including a random intercept for census tract. In sensitivity analyses, we examined the effect of stratification by education and income category in the individual-level analyses. All analyses were conducted in SAS v9.3 [Cary, NC].

## Results

**Figure 1:**
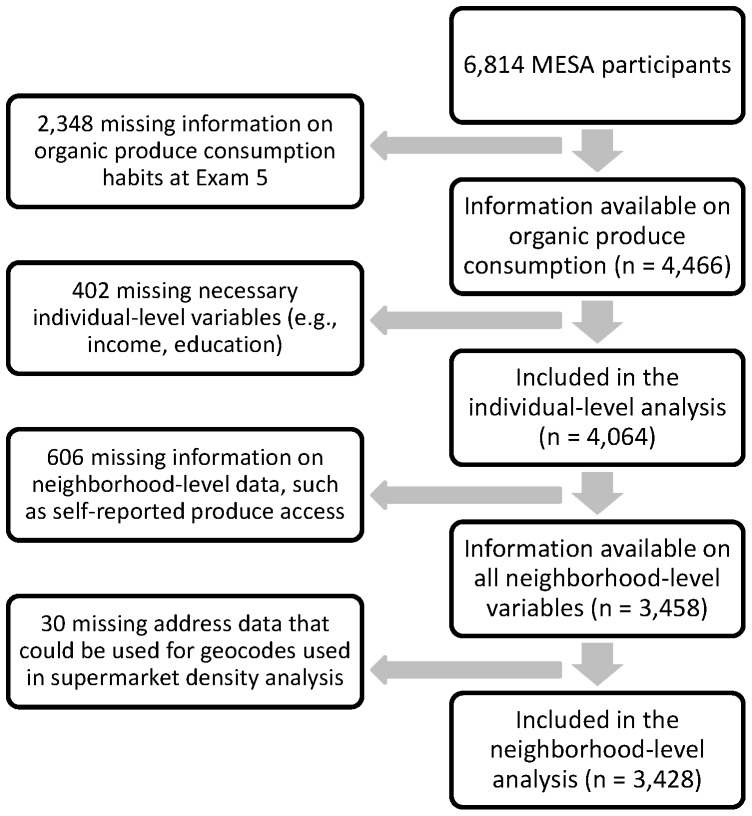
Exclusion criteria and sample sizes for the individual- and neighborhood-level analyses. The largest data loss occurs between study enrollment in 2000–2002 and Exam 5, in which this study occurs, in 2010–2012.

Of the original MESA cohort (n = 6,814), 4,466 (66%) participated in Exam 5 and responded to the questions on organic consumption habits. Complete demographic and socioeconomic data were available on 4,064 participants (see Figure 1). Overall, 204 (5%) reported “often or always” eating both organic fruit and organic vegetables, 1,440 (35%) reported that they “sometimes” ate organic fruit and/or organic vegetables, and 2,420 (60%) “seldom or never” ate organic produce.

### Organic Produce Consumption and Individual-Level Variables

Table 1 shows descriptive individual-level statistics by reported organic consumption habits. In bivariate analyses, organic consumption was significantly more common among women, younger individuals, and those currently employed. Metropolitan area was also significantly associated with organic consumption, as were higher per capita household income and education.

**Table 1:** Demographic and socioeconomic characteristics of the Multi-Ethnic Study of Atherosclerosis cohort at Exam 5 (2010–2012), by organic produce consumption habits.

	Never or rarely consume organic produce		Sometimes, often or always consume organic produce		Bivariate analysis^a^
Total sample (n = 4,064)	2420	60%	1644	40%	
**Gender**					
Female	1213	57%	927	43%	**<0.0001**
Male	1207	63%	717	37%	
**Race/Ethnicity**					
Caucasian	975	58%	710	42%	0.08
Chinese	304	62%	187	38%	
African-American	616	59%	434	41%	
Hispanic	525	63%	313	37%	
**Age**					
45–54	26	39%	40	61%	**<0.0001** b
55–64	750	54%	634	46%	
64–74	764	58%	545	42%	
75–84	690	67%	345	33%	
>85	190	70%	80	30%	
**Marital status**					
Married	1443	59%	1019	41%	0.13
Not married	977	61%	625	39%	
**Metropolitan area**					
Chicago, IL	415	53%	361	47%	**<0.0001**
Winston-Salem, NC	414	63%	245	37%	
New York, NY	398	56%	312	44%	
Baltimore, MD	345	62%	215	38%	
St. Paul, MN	464	67%	225	33%	
Los Angeles, CA	384	57%	286	43%	
**Per capita household income**					
<$14,999	713	65%	382	35%	**<0.0001**
$15,000–24,999	533	65%	293	35%	
$25,000–$34,999	425	60%	287	40%	
$35,000–$44,999	215	54%	185	46%	
>$45,000	534	52%	497	48%	
**Education**					
Less than high school	375	71%	151	29%	**<0.0001**
High school degree	490	70%	206	30%	
Some college	681	57%	521	43%	
Bachelor's degree	441	58%	315	42%	
Graduate degree	433	49%	451	51%	
**Employment Status**					
Unemployed or Retired	1900	60%	1243	40%	**0.03**
Employed	520	56%	401	44%	

a p-values derived from either chi-squared (gender, race, marital status, metropolitan area and employment status, per capita income, education) or log-binomial regression (age).

b The age distribution is shown in categories for display purposes, but was modeled as a continuous variable in a log-binomial regression.

Table 2 shows the associations between individual-level variables and organic consumption in a multivariate log-binomial regression model including all variables with statistically significant bivariate associations. Race/ethnicity was also included because of the importance of this variable in this cohort and to diet in general. After accounting for other individual-level factors, women were more likely to be organic consumers than men (prevalence ratio [PR]: 1.21, confidence interval [CI]: 1.12–1.30, p<0.0001). Chinese participants were less likely than other participants to be organic consumers, though this difference was not large and overall, race/ethnicity did not show a statistically significant effect. Age was highly associated with organic consumption; for every +10-year increment in age, there was a 13% reduction in the likelihood of being an organic consumer.

**Table 2:** Prevalence ratios and 95% confidence intervals for the association between organic food consumption and individual-level demographic and socioeconomic characteristics in adjusted models.

	Prevalence ratio	Confidence Interval	p-value
**Gender**			
Male	Referent		**<0.0001**
Female	1.21	1.12–1.30	
**Race/Ethnicity**			
Caucasian	Referent		0.23
Chinese	0.86	0.75–1.00	
African-American	0.96	0.88–1.06	
Hispanic	0.98	0.87–1.10	
**Age**			
Continuous, per 10 years	0.87	0.84–0.91	**<0.0001**
**Metropolitan area**			
Chicago, IL	Referent		**<0.0001**
Winston-Salem, NC	0.84	0.74–0.95	
New York, NY	1.05	0.94–1.19	
Baltimore, MD	0.86	0.75–0.97	
St. Paul, MN	0.78	0.67–0.89	
Los Angeles, CA	1.13	1.00–1.27	
**Per capita household income**			
<$14,999	Referent		0.06
$15,000–24,999	0.94	0.83–1.06	
$25,000–$34,999	1.03	0.91–1.16	
$35,000–$44,999	1.10	0.96–1.27	
>$45,000	1.10	0.98–1.24	
**Education**			
Less than high school	Referent		**<0.0001**
High school degree	1.05	0.88–1.26	
Some college	1.49	1.27–1.75	
Bachelor's degree	1.39	1.16–1.65	
Graduate degree	1.68	1.42–1.99	
**Employment status**			
Unemployed or Retired	Referent		0.43
Employed	1.03	0.95–1.12	

Metropolitan area was also significantly associated with organic consumption in this cohort: participants living in more the populated cities (Chicago, LA and New York) were more likely to be organic consumers compared to those living in Winston-Salem, Baltimore and St. Paul. Education was found to be an important predictor: comparing the highest and lowest education categories (less than high school compared to graduate school) resulted in a 68% greater likelihood of organic consumption.

Being in the highest income category compared to the lowest (per capital household income of <$14,999 versus >$45,000) was associated with a 10% greater likelihood of organic consumption, but the overall relationship between organic food consumption and per capita household income was not statistically significant (p = 0.06), and higher income was not always associated with greater consumption. For example, participants with per capita household income between $15,000 and $25,000 were less likely to consume organic produce than those in the <$15,000 category. Employment status was not associated with organic consumption in multivariable analyses. Results were not sensitive to stratification of the sample; prevalence ratio point estimates were similar when restricted to just those participants in the lower and higher education and income brackets (data not shown).

### Organic Produce Consumption and the Local Food Environment

Of those participants included in the individual-level analyses, 84% (n = 3,428) consented to MESA Neighborhood and had complete data for the neighborhood-level analyses (see Figure 1). The distribution of demographic and socioeconomic characteristics between this group and those shown in Table 1 is nearly identical. Table 3 shows the frequency of organic consumption among these participants by each measure of the local food environment. In bivariate analyses, whether measured by self-report, supermarket density, or ANS score, participants for whom accessibility was greater were more likely to be organic consumers.

**Table 3:** Frequency of organic food consumption in relationship to measures of the local food environment.

	Never or rarely consume organic produce		Sometimes, often or always consume organic produce		Bivariate analyses^a^
	n	%	n	%	p-value
**Total sample (n = 3,428)**	**2046**	**60%**	**1382**	**40%**	
**Density of supermarkets within 1 mile of residence (by quartile)**					
Quartile 1 (0–0.3 per sq mile)	703	63%	417	37%	**<0.0001**
Quartile 2 (0.3–0.6 per sq mile)	456	60%	302	40%	
Quartile 3 (0.6–1.6 per sq mile)	363	61%	234	39%	
Quartile 4 (1.6–11.8 per sq mile)	524	55%	429	45%	
**Self-report: “A large selection of fresh fruits/vegetables is available in my neighborhood”**					
Disagree or strongly disagree	390	67%	192	33%	**<0.0001**
Neutral	159	59%	109	41%	
Agree or strongly agree	1497	58%	1081	42%	
**Aggregated Neighborhood Survey (by quartile)**					
Quartile 1 (2.8–3.5)	553	65%	304	35%	**<0.0001**
Quartile 2 (3.5–3.8)	546	64%	310	36%	
Quartile 3 (3.8–4.0)	496	58%	361	42%	
Quartile 4 (4.0–4.5)	451	53%	407	47%	

a p-values derived from log-binomial regression with variables specified as continuous.

**Figure 2:**
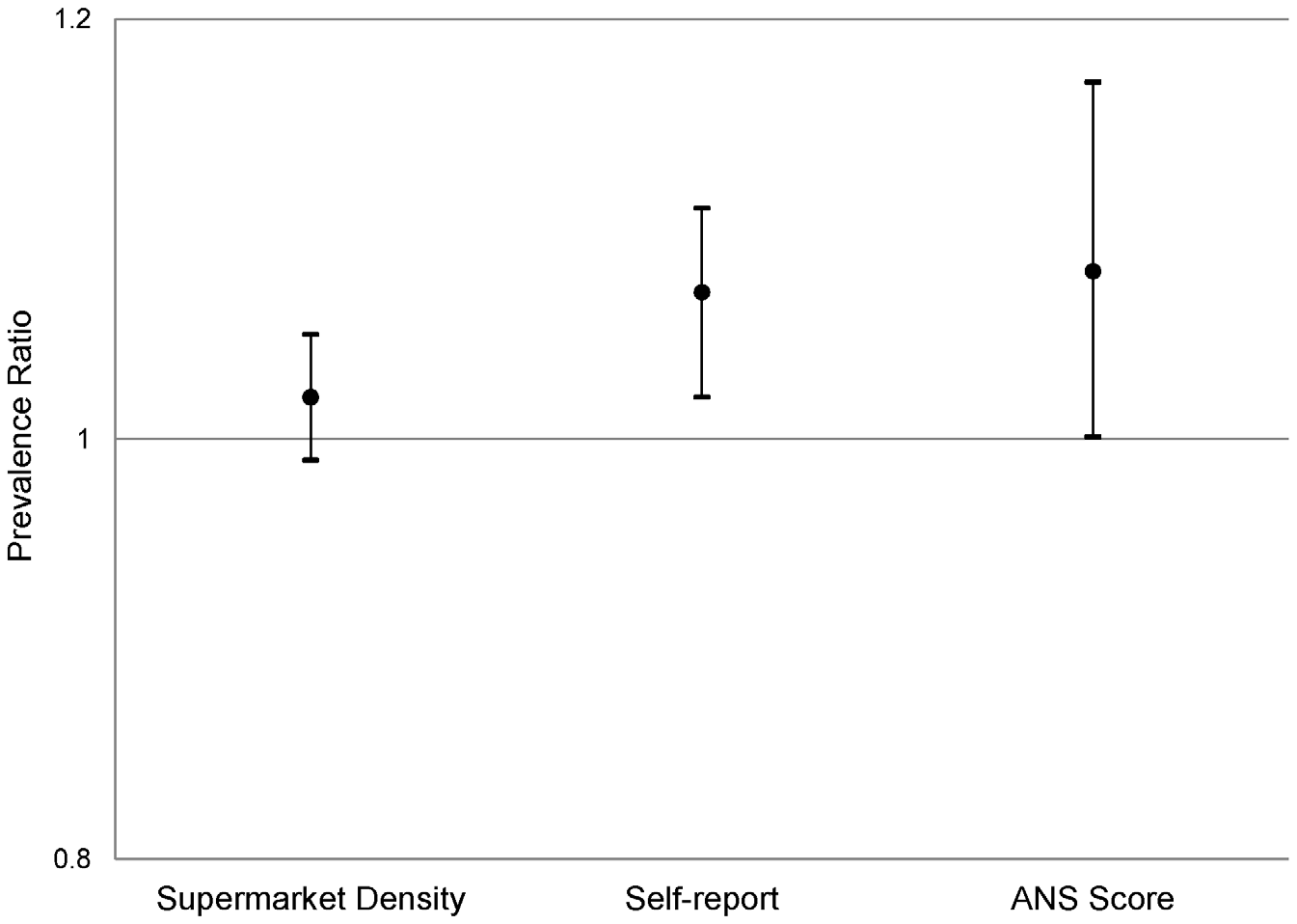
Associations of organic food consumption with neighborhood food accessibility. Food accessibility is estimated by a) density of supermarkets (per increase in one supermarket per mile); b) self-report of fruit and vegetable selection in a participant's neighborhood (per one point increase on the Likert scale); and c) Aggregated Neighborhood Survey (per interquartile difference, represented by a 0.5 increase on the Likert scale). Models are adjusted for sex, age, education, income, metropolitan area, and race/ethnicity.

This association remained in fully adjusted models as well (Figure 2). After adjusting for individual-level variables, self-reported produce availability within a participant's neighborhood was positively associated with organic consumption; each unit increase on the Likert scale, was associated with a 7% greater likelihood of eating organic food (PR: 1.07, CI: 1.02–1.11, p = 0.002). The ANS score analysis also suggested an effect of local food environment on organic consumption; the likelihood of being an organic consumer was 8% higher per interquartile change in score (0.5 units) (PR: 1.08, CI: 1.00–1.17, p = 0.05). Inclusion of a random intercept for each census tract did not substantially modify estimates or standard errors in any of the three models. The GIS-based supermarket density measure was not significantly associated with organic consumption after control for individual-level variables, though the direction of the effect was unchanged (PR: 1.02, CI: 0.99–1.05, p = 0.16). All individual factors associated with organic consumption remained significant with the inclusion of the measures of the local food environment.

### Frequent Organic Consumers

While the primary analysis aimed to understand the factors associated with the decision to consume organic produce at least occasionally, this secondary analysis explored the question of whether individual and local food environment factors were associated with more frequent organic produce consumption. Here, the definition of organic consumers was restricted to those who reported that the fruit and vegetables they ate were “often or always” organic. In general, the relationships between organic produce consumption and individual-level factors were similar to those reported in the primary analyses. In fully adjusted models of individual factors, female gender, younger age, more urban metropolitan areas, and higher levels of education were all significantly associated with “often or always” consuming organic produce, as was the case in the primary analysis. Race/ethnicity and marital status were not significantly associated with organic produce consumption. Per capita household income was significantly associated with “often or always” consuming organic produce (p = 0.04), but the relationship was not linear. Instead, the lowest and highest income groups were more likely to report that they “often or always” consumed organic produce, and the middle income groups were significantly less likely to be frequent organic produce consumers.

However, in contrast to the results of the primary analysis, the local food environment was not associated with the decision to “often or always” consume organic produce. Though density of supermarkets within 1 mile of the residence remained strongly associated with organic produce consumption in bivariate analyses (p = 0.0002), aggregated neighborhood survey and self-report of accessibility were no longer significantly associated (p = 0.09 and p = 0.17, respectively). Further, once individual-level variables were accounted for in a fully adjusted model, no significant relationship was found between “often or always” consuming organic produce and any of the measures of the local food environment (supermarket density: OR = 1.05, CI = 0.95–1.17; self-report: OR = 1.00, CI = 0.85–1.17; ANS Score: OR = 1.01, CI = 0.52–1.98).

## Discussion

To our knowledge, this is the first study to examine the associations of both individual and neighborhood characteristics and organic food consumption. We found that both are associated with this dietary choice. Women, younger individuals, those with higher education, and those living in more urban areas were more likely to consume organic produce. Neither race/ethnicity nor per capita household income was strongly associated with organic produce consumption. We found that characteristics of the local food environment, such as produce availability, were associated with the decision to consume organic produce at least occasionally.

### Individual-Level Findings

Organic food consumption is increasing; consistent with our findings, several studies over the past decade have reported that 40 to 50% of individuals and households purchase organic food at least occasionally [23]–[27]. However, the specific factors associated with organic food consumption have not been well understood, as early studies painted contradictory pictures of the socioeconomic status and demographics of organic food consumers.

Organic food consumption has been found to be associated, variously, with higher education [28]–[29], lower education [30], or not associated with education at all [31]. Results were also mixed for the relationship between income and organic food; some studies observed consumers with high incomes to have less tolerance for food with blemishes and to be less likely to purchase organic food [29], while others found people with higher income to be more likely to make organic purchases [28], [30], and others found no association [31]–[32]. Findings with respect to age and ethnicity were also inconsistent; in fact, the only demographic attribute to be reliably associated was gender, with women purchasing more organic food than men [28], [30]–[31]. However, all of these studies employed convenience samples, and typically included people who were already shopping at either food cooperatives or at expensive specialty grocers, missing substantial segments of the general population. More recent research has capitalized on Nielsen Consumer Panel studies, in which thousands of American households are provided handheld scanners to scan each item they purchase [24], [27], [33]. These studies have consistently found higher income and education to be associated with purchasing organic food, but age and ethnicity have continued to show inconsistent effects.

Our results are consistent with previous research showing that women purchase organic food more frequently than men, and with the Nielsen Consumer Panel studies' observation that higher education is associated with more organic food purchasing. In MESA, older participants were less likely than younger participants to consume organic food. However, since all participants in this study were aged 45 and older, this result could also be consistent with a “U-shaped” relationship between age and organic consumption, in which middle aged people are more likely to consume organic food than both younger and older individuals, as other researchers have suggested [34].

The relationship between per capita household income and organic produce consumption was sensitive to adjustment for other individual level variables, and to the categorization of organic consumers. In bivariate analyses, self-report of either “sometimes” or “often or always” consuming organic produce was strongly associated with income category, but the strength of this relationship was attenuated by adjustment for other individual level factors. When restricted to individuals who “often or always” consumed organic produce, the relationship with income was decidedly non-linear; individuals with the lowest and highest per capita household incomes were more likely to report frequent consumption of organic produce.

To our knowledge, this is the first study to examine geographical differences in organic food consumption; we found participants in more populated cities (Los Angeles, Chicago, and New York) to consume more organic food than those in less densely populated regions (St. Paul, Winston-Salem and Baltimore). This difference may also be related to the local food environment and food access; more research is needed to fully understand the relationship between organic food consumption and urbanicity.

### Local Food Environment Findings

Over the past decade, the US obesity epidemic – and in particular, disparities in obesity prevalence – has led the public health community to think much more broadly about factors influencing diet. No longer are dietary motivations understood only in the framework of individual lifestyle choices. Instead, the food environment has been increasingly recognized as important to diet [15], [18], [35]–[39], and the results of this study are consistent with the idea that this environment influences a variety of dietary choices.

While this is the first study to explicitly investigate the relationship between the local food environment and organic consumption, it is not the first study to look at factors beyond demographics and socioeconomics on this dietary choice. Zepeda and colleagues explored the motivations behind organic food consumption in a national survey of nearly 1,000 US adults [23], [40]. When variables related to food beliefs and shopping habits were considered, organic food consumption was not found to be associated with direct economic variables, such as household income or weekly food expenditures. Instead, the important factors in choosing organic food included both personal beliefs and opportunity, where opportunity was defined as shopping at food venues where organic food was more likely to be available. This represents a different approach to exploring the influence of food accessibility on organic consumption, but the results are consistent with our finding that access may play an important role in the decision to at least occasionally consume organic foods. The results from our secondary analysis support the notion that personal beliefs may matter, perhaps particularly for those who often or always consume organic food. This relatively small group of people may be willing to go out of their way to make this dietary choice, even if produce and supermarkets are not readily available in their neighborhoods.

### Limitations

A primary limitation of this study was the lack of a direct measure of organic food availability. Instead, we employed supermarket density and both self-report and community perception of availability of produce and healthy food as proxies for organic food availability. The USDA's 2009 report, “Marketing US Organic Foods: Recent Trends from Farms to Consumers” shows that sales of organic food from conventional supermarkets and groceries now account for 46% of the total organic market share, with natural-products retailers and direct markets each accounting for another 44% and 10%, respectively [41]. This report also states that organic food is now available in more than 80 percent of all supermarkets. Given this high proportion of supermarkets in which organic food is available, we believe that it is reasonable to assume that areas with more supermarkets are also more likely to provide greater access to organic food. In addition, organic produce is more likely to be available in areas with a greater selection of produce in general. However, further research including more specific measures of organic food availability is warranted.

Recent literature has shown that subjective and objective measures of the local food environment do not always agree [42]–[43], and so we investigated three complementary measures of the local food environment, each with strengths and weaknesses. Supermarket densities are the most objective of the three but rely on the assumption that supermarkets offer organic foods. Further, the use of supermarket density within a straight-line distance neglects actual travel patterns along road networks and further assumes that people reliably shop at supermarkets near their homes. Recent research by Drewnowski et al. suggests that this assumption may not accurately reflect actual shopping patterns [44]. Self-reports reflect each individual's perceptions but their interpretation is affected by the possibility of same source bias, which may arise when using self-reports of the food environment if a person's behavior affects their reported perceptions of access to healthy foods. The strength of aggregate survey measure is that averages of multiple respondents are likely to eliminate the influence of individual subjectivities and eliminate the possibility of same–source bias. However, it may not accurately reflect access for a particular participant. The consistency of associations across the three types of measures increases confidence that the local food environment plays a role in organic consumption habits.

We chose to focus on organic produce rather than other food types. This was intended to be consistent with previous studies evaluating the relationship between consumption of organic food and organophosphate pesticide exposure [4]. Over 33 million pounds of organophosphates are applied annually in the US – more than any other class of insecticides – and their metabolites are found in the urine of 94% of the US population [45]. These compounds are registered for use on a wide variety of fruits and vegetables, but are not widely used in the production of meat or dairy [46]. We do not expect that this decision had a large impact on our results, as recent USDA research shows that US retail sales of organic fruits and vegetables are larger than all other organic food categories combined [41]. However, it is worth noting that there are several other categories of organic food not considered here.

## Conclusions

This study demonstrates that both individual- and neighborhood-level characteristics are associated with the decision to consume organic produce at least occasionally, and provides further evidence of the impact of food access on dietary choices. While previous research has shown that healthy food environments are associated with healthy diets, this is the first study to explore the relationship between the local food environment and organic food consumption. While it remains unclear whether or not there is a health benefit to eating organic food, there is growing evidence that consumption of organic food can reduce pesticide exposure and that, at least for some segments of the population, even low levels of pesticide exposure may have health effects. There are also other reasons that people may choose to eat organic food, including concerns for farmworker safety and ecological health. Allowing everyone equal ability to make this choice may present yet another argument for leveling the playing field of food accessibility.
